# Primary hyperparathyroidism and recurrent ventricular tachyarrhythmia in a patient with novel *RyR2* variant but without structural heart disease

**DOI:** 10.1002/ccr3.2363

**Published:** 2019-08-23

**Authors:** Christian Møller Pedersen, Lars Rolighed, Torben Harsløf, Henrik Kjærulf Jensen, Jens C. Nielsen

**Affiliations:** ^1^ Department of Cardiology Aarhus University Hospital Skejby Aarhus Denmark; ^2^ Department of Cardiology Regionl Hospital West Jutland Herning Denmark; ^3^ Department of Otolaryngology Aarhus University Hospital Skejby Aarhus Denmark; ^4^ Department of Endocrinology and Internal Medicine Aarhus University Hospital Skejby Aarhus Denmark

**Keywords:** hypercalcemia, parathyroid adenoma, primary hyperparathyroidism, ventricular tachyarrhythmia

## Abstract

It is important to consider calcium and parathyroid hormone levels in patients with recurrent VT/VF without any obvious cause of arrhythmia. In similar cases to gain rhythm control using isoprenaline and do comprehensive molecular‐genetic. Diagnosis and surgery in case of parathyroid adenoma may be needed to obtain definite arrhythmia control.

## 1 INTRODUCTION

The parathyroid gland secretes parathyroid hormone (PTH), which is the main regulator of the calcium, phosphate, and bone metabolism. Primary hyperparathyroidism (PHPT) due to increased secretion of PTH from the parathyroid glands arises from an adenoma or hyperplasia of the glands and leads to hypercalcemia and hypophosphatemia.^1^ The clinical investigation of PHPT evaluates symptoms of hypercalcemia such as fatigue, thirst, constipation, confusion, and depression and explores the presence of complications from PHPT such as renal insufficiency, kidney stones, or osteoporosis. Hypercalcemia has been described to cause ECG changes and ventricular ectopic beats, but only very rarely sustained cardiac arrhythmias.^2^, ^3^

## 2 CASE REPORT

We report a case of recurrent life‐threatening ventricular tachyarrhythmia in a 62‐year‐old male patient with a history of hypertension and gout. Eighteen months prior to admission, the patient underwent echocardiography and coronary angiography due to stable angina pectoris. The echocardiography showed a normal left ventricular function and no indication of structural heart disease. The coronary angiogram showed moderate diffuse atherosclerosis without any significant stenosis.

The patient was admitted on 6 December 2017 after witnessed OHCA (out‐of‐hospital‐cardiac‐arrest) at home. The patient had not complained of any symptoms before the event. Paramedics and anesthesiologist arrived 8 minutes after cardiac arrest. Upon rhythm analysis, the initial heart rhythm was recurrent ventricular fibrillation (VF) and the patient needed DC‐defibrillated twice to establish stable sinus rhythm (SR). He was stabilized hemodynamically, and the ECG showed SR without any signs of myocardial ischemia, narrow QRS complexes, and with the corrected QT interval (QTc) within normal range. Acute coronary angiography revealed no significant stenosis or occlusion in the coronary arteries, and no revascularization was indicated. Echocardiography revealed no pathology and a normal left ventricular function. Acute CT of the brain, thorax, and abdomen was likewise without pathology. The patient was taken to the intensive care unit and underwent therapeutic hypothermia postcardiac arrest with cooling to 33 degrees celsius for 24 hours.

Two days after admission, and after reestablishing normothermia, EEG (electroencephalography) and SEP (somatosensory evoked potentials) were performed, showing no signs of irreversible brain damage, but the EEG did show 2½‐3 Hz sharp‐wave complexes indicating nonconvulsive epileptic status and the patient started treatment with levetiracetam (1500 mg × 2), valproat (750 mg × 2), and clonazepam (1 g × 4). A new EEG 3 days later showed no signs of epilepsy, and the antiepileptic treatment was discontinued.

At admission, biochemistry showed normal levels of potassium, sodium, phosphate, and magnesium. In accordance with national Danish guidelines on treatment of hypercalcemia (www.Endocrinology.dk), the patient was kept well‐hydrated with a minimum of 3 L of isotonic saline/d. Calcium level was elevated above normal to 2.97 (2.20‐2.55) mmol/L, ionized calcium was 1.60 (1.18‐1.32) mmol/L, and PTH was 28.8 (1.6‐6.9) pmol/L). The patient therefore received injection of Calcitonin 600IE. Marginal increase in Troponin T to 163 (<14) ng/L was observed on December 7th.

Two days later, PTH was 55.5 pmol/L, calcium was 3.21 mmol/L, and ionized calcium 1.69 mmol/L. Another dose of Calcitonin 600IE was administered.

During the next 7 days, the patient was stable in the ICU and PTH and calcium levels remained unchanged, though still elevated.

**Figure 1:**
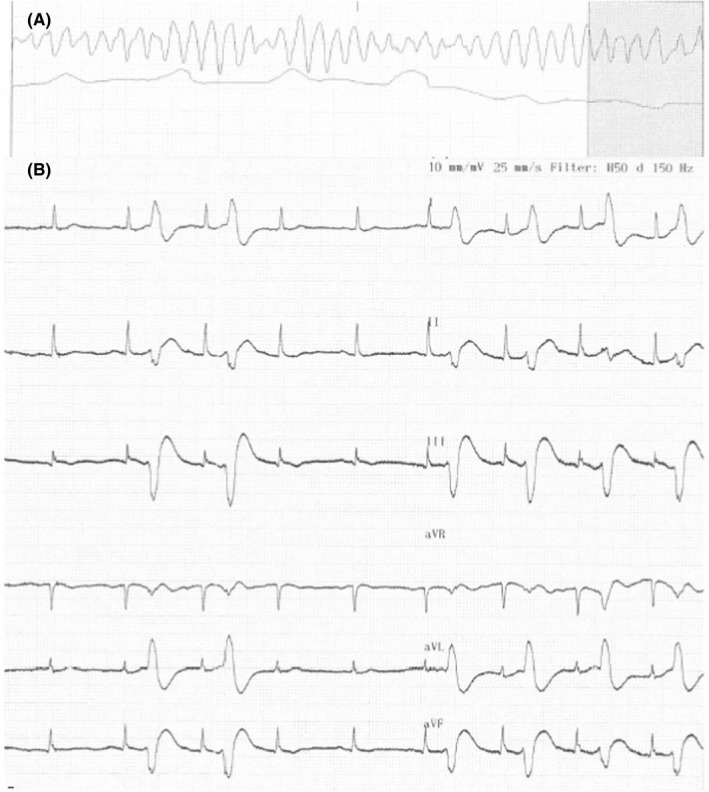
A, Ventricular tachyarrhythmia when the patient had IHCA. B, Multiple ectopic beats, very close to the T‐wave leading to ventricular tachyarrhythmia

On December 17th, the patient had IHCA (in‐hospital cardiac arrest). The initial rhythm was ventricular tachycardia (VT) degenerating to VF (Figure 1A). DC‐defibrillation was performed 6× because of recurrent VF. Defibrillations were effective in establishing SR. Adrenalin (1 mg × 2 IV), Amiodarone (300 mg IV), and Magnesium (10 mmol IV) were administered according to guidelines. On ECG monitoring, it was evident that multiple ventricular ectopic beats were present, eliciting both nonsustained and sustained VT (Figure 1B) degenerating to VF. After arrival in the cardiac catheterization laboratory, the patient was stabilized on intravenous infusion with Isoprenaline to maintain SR with rate of 100‐110 beats per minute (bpm) and to prevent ectopic ventricular beats leading to ventricular tachyarrhythmia. Acute coronary angiography was performed, showing no culprit lesion. Acute bedside echocardiography revealed no pathology. The blood analysis showed potassium and sodium within normal ranges, significantly decreased the level of phosphate 0.28 (0.71‐1.22) mmol/L), increased the level of magnesium 1.25 mmol/L, calcium of 2.94 mmol/L, and highly elevated PTH of 170.7 pmol/L.

**Figure 2:**
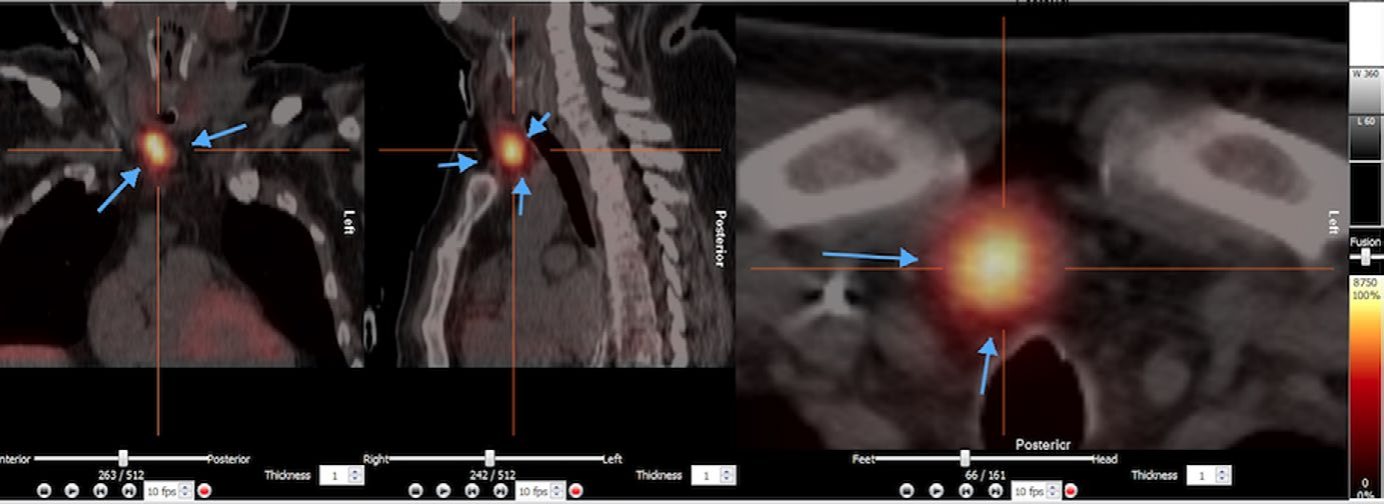
^99M^Tc‐sestamibi‐single photon emission computed tomography (SPECT) scan, showing a large adenoma of the right lower parathyroid gland

**Figure 3:**
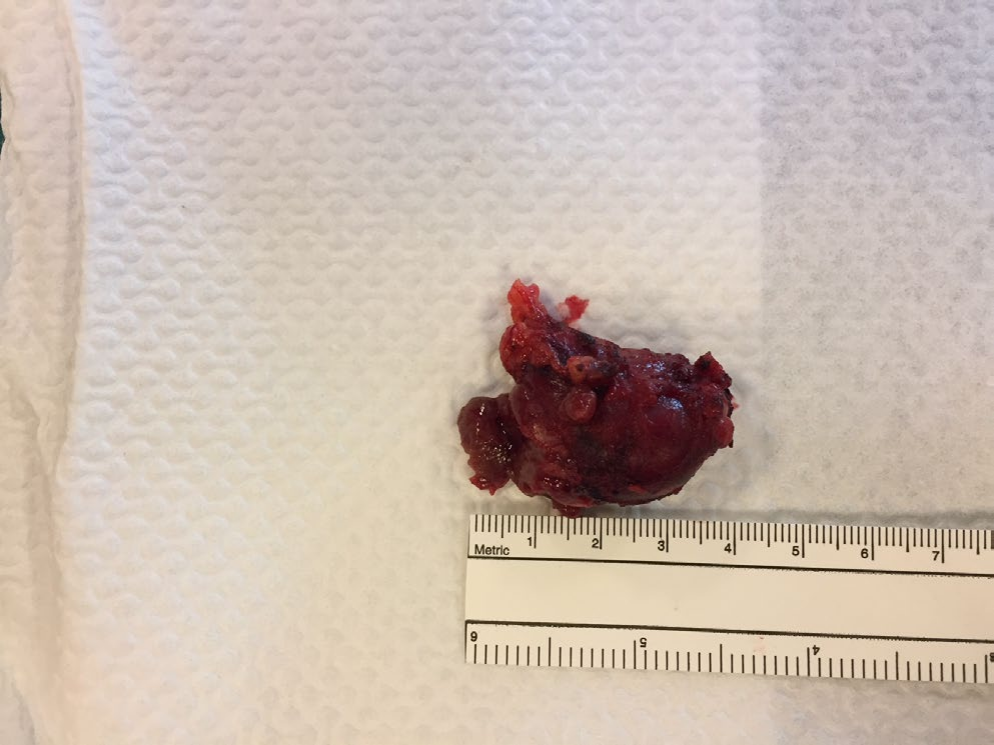
Adenoma of the parathyroid gland remove by surgery (4 × 2 × 1.5 cm)

**Figure 4:**
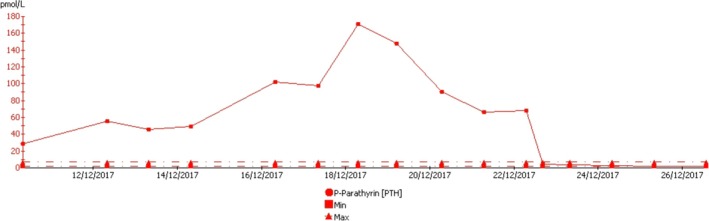
Blood levels of PTH in course of time from admittance to 4th postoperative day

The patient remained stable on Isoprenaline‐infusion without ectopic ventricular activity and without VT and VF. We administered Cinecalcet (increasing sensitivity to extracellular calcium in the calcium‐sensitive receptor (CaSR) leading to a downregulation of PTH) at a dose of 60 mg twice daily. On the following day, ultrasound scan of the neck was performed, showing no clear signs of pathology. Two days later, the patient underwent ^99m^Tc‐sestamibi‐single photon emission computed tomography (SPECT) scan, showing a large adenoma of the right lower parathyroid gland (Figure 2). Surgery was performed the following day with removal of a 4 × 2 × 1.5 cm large adenoma (Figure 3), resulting in rapid normalization of the PTH level (Figure 4) and a decrease in calcium level. Because of the prolonged effect of Cinacalcet, calcium substitution was needed in the acute setting and is going to be continued longterm to prevent significant hypocalcemia. After normalization of both PTH and calcium levels, magnetic resonance imaging (MRI) scan of the heart was performed showing no signs of pathology. The patient was monitored in hospital for another 7 days without any recurrent arrhythmia.

Despite surgical removal of the adenoma resulting in normalized blood calcium levels, the patient was offered implantation of a single‐chamber (VVI) ICD to yield protection from sudden death in case of future ventricular tachyarrhythmia. After 8 months of follow‐up, we observed no recurrent arrhythmia.

**Figure 5:**
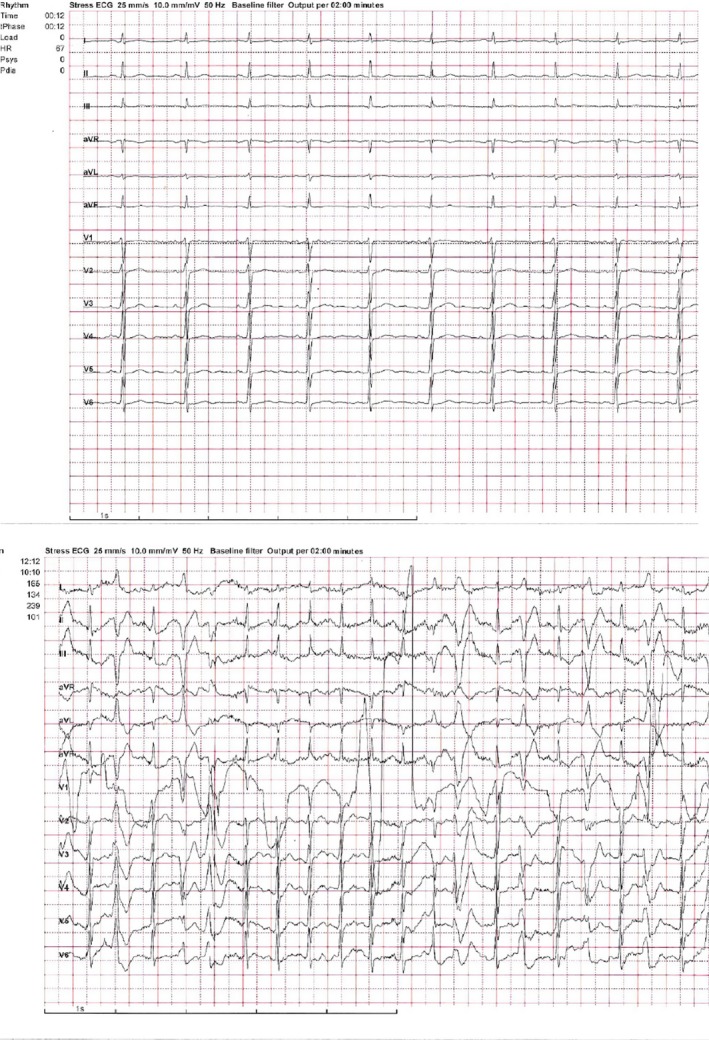
Bidirectional PVC during exercise test, baseline and 165 watts

After discharge, our patient underwent exercise test in which bidirectional premature ventricular contractions were observed, as typical for catecholaminergic polymorphic ventricular tachycardia (CPVT) (Figure 5).

During follow‐up, comprehensive molecular‐genetic testing (MOMA heart panel v3. For a full list of genes sequenced, see http://moma.dk/genetic-analysis) revealed a missense variant in the Ryanodine receptor 2 (*RyR2)* gene:c.8741C > T p.(Thr2914Met) with minor allele frequency i gnomAD database (2.0.2) 0.0018% in non‐Finnish Europeans. Moderate conserved nucleotide and highly conserved aminoacid. In silico programs, SIFT and Mutation Taster, predict it to be disease‐causing and PolyPhen, possible disease‐causing.

## 3 DISCUSSION

We present a patient with repetitive life‐threatening ventricular tachyarrhythmia associated with rapidly increasing PTH level and moderate hypercalcemia, where arrhythmia control was obtained with subacute removal of a large parathyroid adenoma. Acutely, Isoprenaline‐infusion was helpful for rhythm stabilization. Our patient was known to have hypertension, but he had no structural heart disease, no significant coronary artery disease, and no other electrolyte disturbances.

Previously, only a few case reports describing a relationship between PHPT, hypercalcemia, and malignant ventricular tachyarrhythmia have been published. In 1988, Chadli et al described ventricular bigeminy associated with hyperparathyroid crisis,^4^ but without more malignant ventricular arrhythmia. It was not described whether structural heart disease was present. In most previously reported cases, patients were known with conditions associated with increased risk of ventricular tachyarrhythmia such as dilated cardiomyopathy,^5^, ^6^ prior myocardial infarction with left ventricular ejection fraction of 30%,^7^ significant general atherosclerosis with previous history of stroke^8^ or hypercalcemia combined with severely decreased level of potassium.^5^, ^9^ Both monomorphic VT^5^, ^10^ and polymorphic VT/VF have been described^8^, ^9^ with severe PHPT. In 2000, Chang et al^10^ described a case in a patient with no history of heart disease, normal left ventricular function, and normal coronary arteries, where the clinical monomorphic VT could be induced during electrophysiological study after infusion of Calcium Gluconate.

The mechanisms causing ventricular arrhythmia are not completely clear. In relation to hypercalcemia, the predominant alteration in the cardiomyocytes is a shortening in the action potential duration leading to decreased ventricular conduction velocity and shortening of the refractory period. These changes at the cellular level correlates with prolonged QRS duration and an shortening of the QT interval in the ECG, most often leading to bradycardia and slowing of the AV‐conduction.^3^ However, in PHPT highly elevated PTH may in addition exert a direct effect on the cardiomyocytes. Experimental data suggest that PTH has a positive inotropic and chronotropic effect mediated by enhanced calcium influx into the cardiomyocyte.^11^, ^12^ The shorter refractory period and the decreased ventricular conduction velocity facilitate reentry circuits to occur, but the combination of elevated PTH and hypercalcemia may also trigger ventricular ectopic beats and less organized ventricular tachyarrhythmia.

In some of the patient cases reported, the plasma level calcium was very high,^5^, ^8^ while in others only moderately increased.^4^, ^9^, ^10^ In our patient, the first blood analysis showed moderate hypercalcemia although PTH level was not severely elevated 28.8 pmol/L (1.6‐6.9). Despite treatment with Calcitonin, the patient had cardiac arrest a few days later with similar blood calcium level, but with a severely elevated PTH 170.7 pmol/L (1.6‐6.9), suggesting that PTH per se may contribute to arrhythmia, likely by influencing the action potential of the cardiomyocytes and the vulnerability for electrolyte disorders. PHPT is a common disease with a yearly incidence of 16 pr 100.000 in Denmark.^13^ Mainstay in the treatment of PHPT is surgery, and surgery is normally indicated in patients <50 years of age or with moderately elevated calcium, established osteoporosis, or renal stones or failure.^14^ A number of retrospective and epidemiological studies show that PHPT is associated with increased mortality from cardiovascular disease.^15^, ^16^ The effect of surgery on mortality, however, is less clear,^17^ indicating that both mortality, and PHPT may be due to a common confounding factor. Four prospective studies comprising more than 400 patients have evaluated the clinical consequences of PHPT either with or without surgery. None of the studies were specifically designed to investigate arrhythmia, but no cases of such event or sudden death were mentioned.^18^, ^19^, ^20^, ^21^ Accordingly, current guidelines on the treatment of PHPT do not consider known cardiovascular disease as an indication for surgery for patients with PHPT.^14^ Taken together, we therefore believe that ventricular arrhythmia in PHPT is a very rare event. The reason that our case presented with ventricular arrhythmia may be due to the *RyR2* variant found during molecular‐genetic testing. The *RyR2* variant:c.8741C > T p.(Thr2914Met) is novel and predicted possible pathogenetic. Given the clinical phenotype observed in our patient (bidirectional PVCs during exercise test), it is likely a mild gain‐of‐function *RyR2* mutation. The cause of the CPVT‐like phenotype in our patient may be a combination of this *RyR2* variant and hypercalcemia, which would be expected to increase calcium loading to the cells and eventually to the sarcoplasmic reticulum resulting in store‐overload‐induced calcium release (SOICR) during physical and emotional stress. The *RyR2* variant may reduce the SOICR threshold.^22^

The case presented is a reminder to consider plasma calcium and PTH levels in patients with recurrent VT/VF where no other obvious cause of arrhythmia is found and in similar cases to make comprehensive molecular‐genetic testing in relevant heart genes. In similar cases, isoprenaline may be used in the acute setting to gain rhythm control. Accelerated diagnosis and surgery in case of parathyroid adenoma may be needed to obtain definite arrhythmia control.

## CONFLICT OF INTEREST

None declared.

## AUTHOR CONTRIBUTIONS

Dr Christian Møller Pedersen PhD MD: Initial diagnostics and treatment of the patient and case report manuscript. Dr Lars Rolighed PhD MD: Surgery treatment of the patient and case report manuscript. Dr Torben Harsløf PhD MD: Case report manuscript. Dr Henrik Kjærulf Jensen DMSc PhD MD: Genetic analysis and case report manuscript. Prof. Jens C Nielsen DMSc PhD MD: Initial diagnostics and treatment of the patient. Case report manuscript.
